# Supplementary material for: Stage-specific epigenetic regulation of CD4 expression by coordinated enhancer elements during T cell development
Source: Nat Commun. 2018 Sep 5;9:3594. doi: 10.1038/s41467-018-05834-w (PMC6125341; doi:10.1038/s41467-018-05834-w)
Supplement: Supplementary file 1 — Supplementary Information [file 41467_2018_5834_MOESM1_ESM.pdf]

Stage-specific epigenetic regulation of CD4 expression by coordinated enhancer elements during T cell development

Issuree *et al.* , 2018

Gating strategy for thymocytes

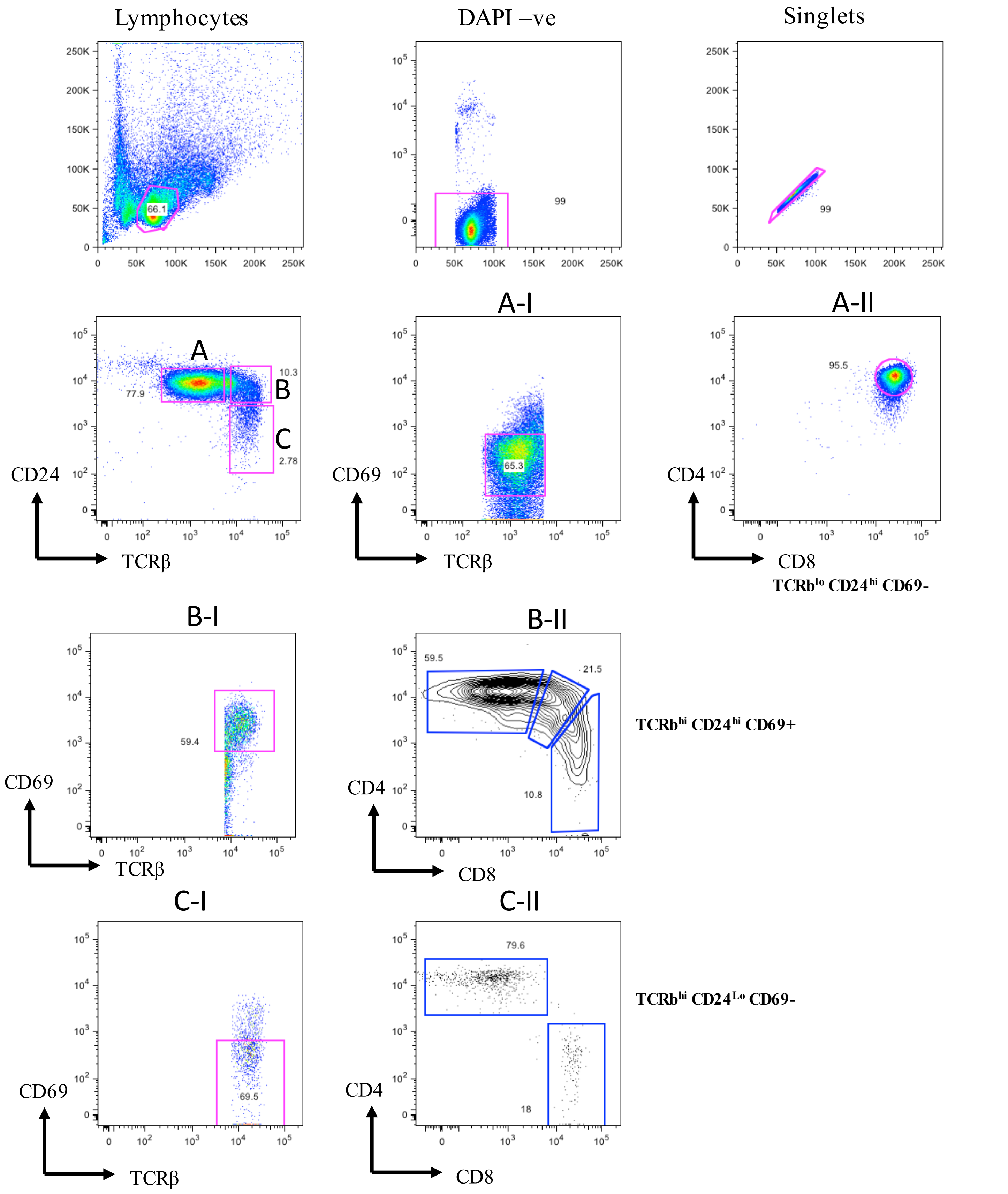

Supplementary Figure 1. Gating strategy to define different stages of αβ T cell maturation in the thymus. Thymocytes isolated from mice were gated on live and single cell populations, followed by gating into 3 separate populations based on CD24 and TCRβ expression. Pre-selected thymocytes were further defined as TCRβ^{lo}CD24^{hi}CD69- (gates A-AII), recently selected thymocytes were TCRβ^{hi} CD24^{hi} CD69^{+} (gates B-BII) while mature thymocytes were TCRβ^{hi}CD24^{lo}CD69- (Gates C-CII).

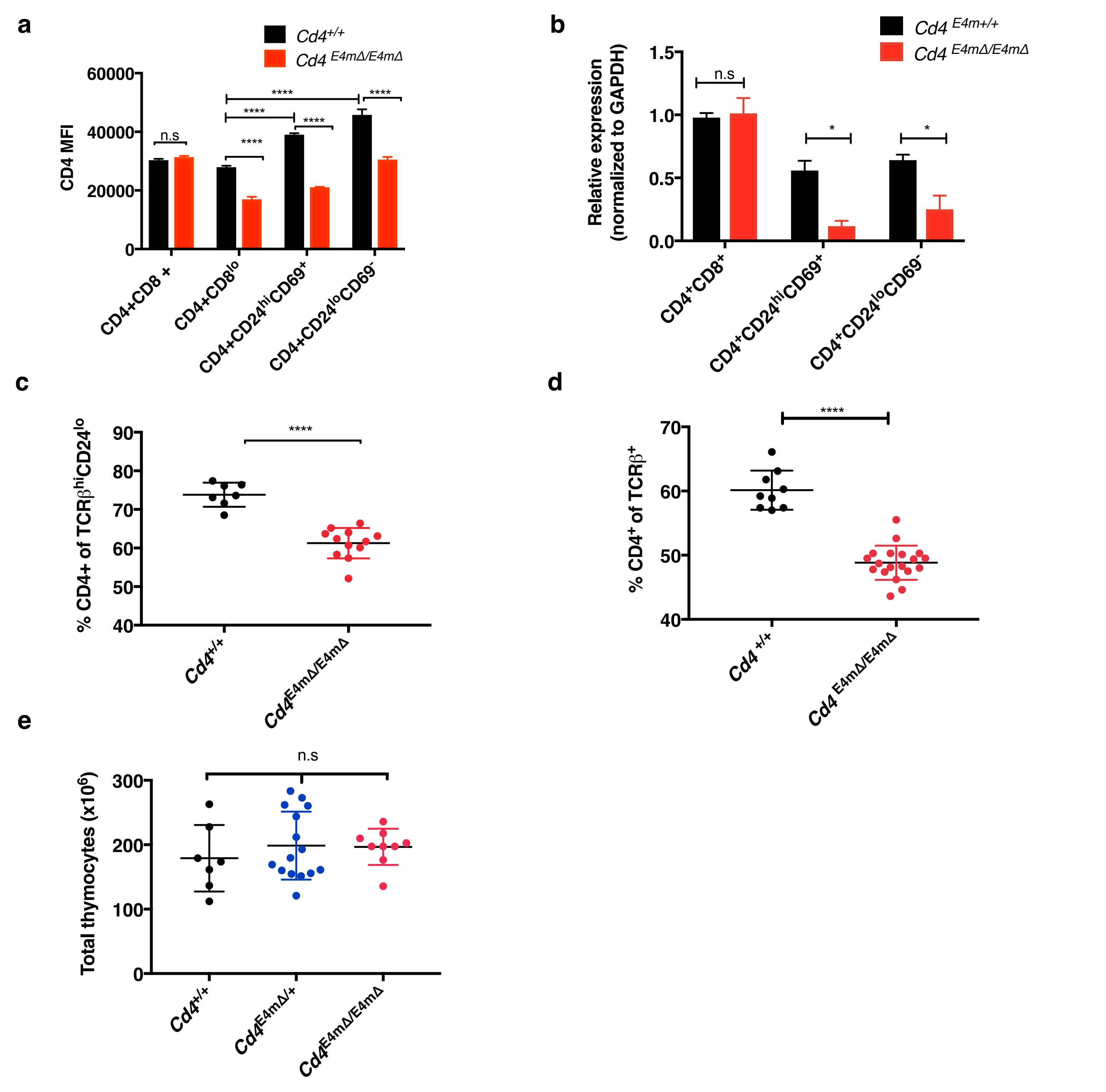

Supplementary Figure 2. E4m regulates *Cd4* expression in recently selected and mature CD4^{+} T cells. **(a)** Mean fluorescence intensity (MFI) of CD4 expressed by indicated subsets of cells from WT and *Cd4* $^{E4m\Delta/E4m\Delta}$ littermate mice. Mean $\pm$ SD. ***p<0.001, ****p<0.0001(unpaired student *t* test) and data is representative of 3 experiments, with 2 mice in each group per experiment. **(b)** qPCR analysis of mature *Cd4* mRNA levels in the indicated subsets of cells from WT and *Cd4* $^{E4m\Delta/E4m\Delta}$ littermates. Mean $\pm$ SD. *p<0.05, (unpaired student *t* test); data from 3 mice in each group. **(c)** Frequency of CD4 SP cells among TCRβ^{hi}CD24^{lo}CD69^{-} thymocytes in WT and *Cd4* $^{E4m\Delta/E4m\Delta}$ littermates. Mean $\pm$ SD. ****p<0.0001 (unpaired student *t* test), with n $\geq$5 in each group. **(d)** Frequency of CD4^{+} T cells among TCRβ^{+} T cells isolated from the spleen and lymph nodes of WT and *Cd4* $^{E4m\Delta/E4m\Delta}$ littermate mice. Mean $\pm$ SD. ****p<0.0001(unpaired student *t* test), with n $\geq$5 in each group. **(e)** Total number of thymocytes in WT, *Cd4* $^{E4m\Delta/+}$ and *Cd4* $^{E4m\Delta/E4m\Delta}$ mice. Experiments are combined from 3 independent analyses.

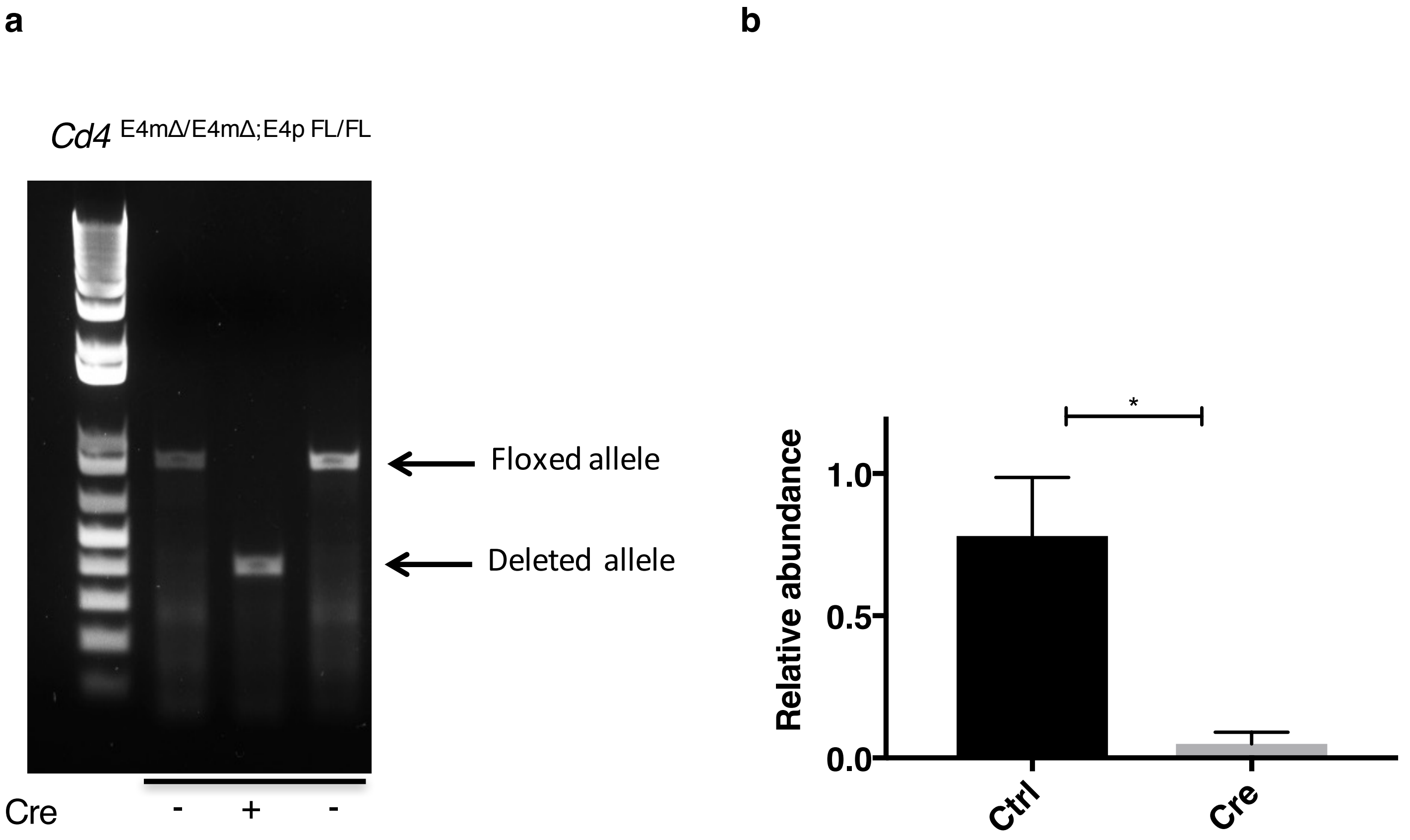

Supplementary Figure 3. E4m and E4p are partially redundant in regulating CD4 expression following positive selection. (a) Analysis of PCR-amplified bands showing excision of the E4p floxed allele upon transduction of naïve $CD4^+$ T cells from *Cd4* $^{E4m\Delta/E4m\Delta;\ E4pFL/FL}$ mice with MSCV Cre-IRES-GFP or control vector. GFP^+ cells were sorted 96 h post transduction and genomic DNA was extracted for PCR analysis. **(b)** RT-qPCR analysis of genomic DNA shown in (a). Data were normalized to expression of *Cd4* exon 1, with 2 replicates for each group.

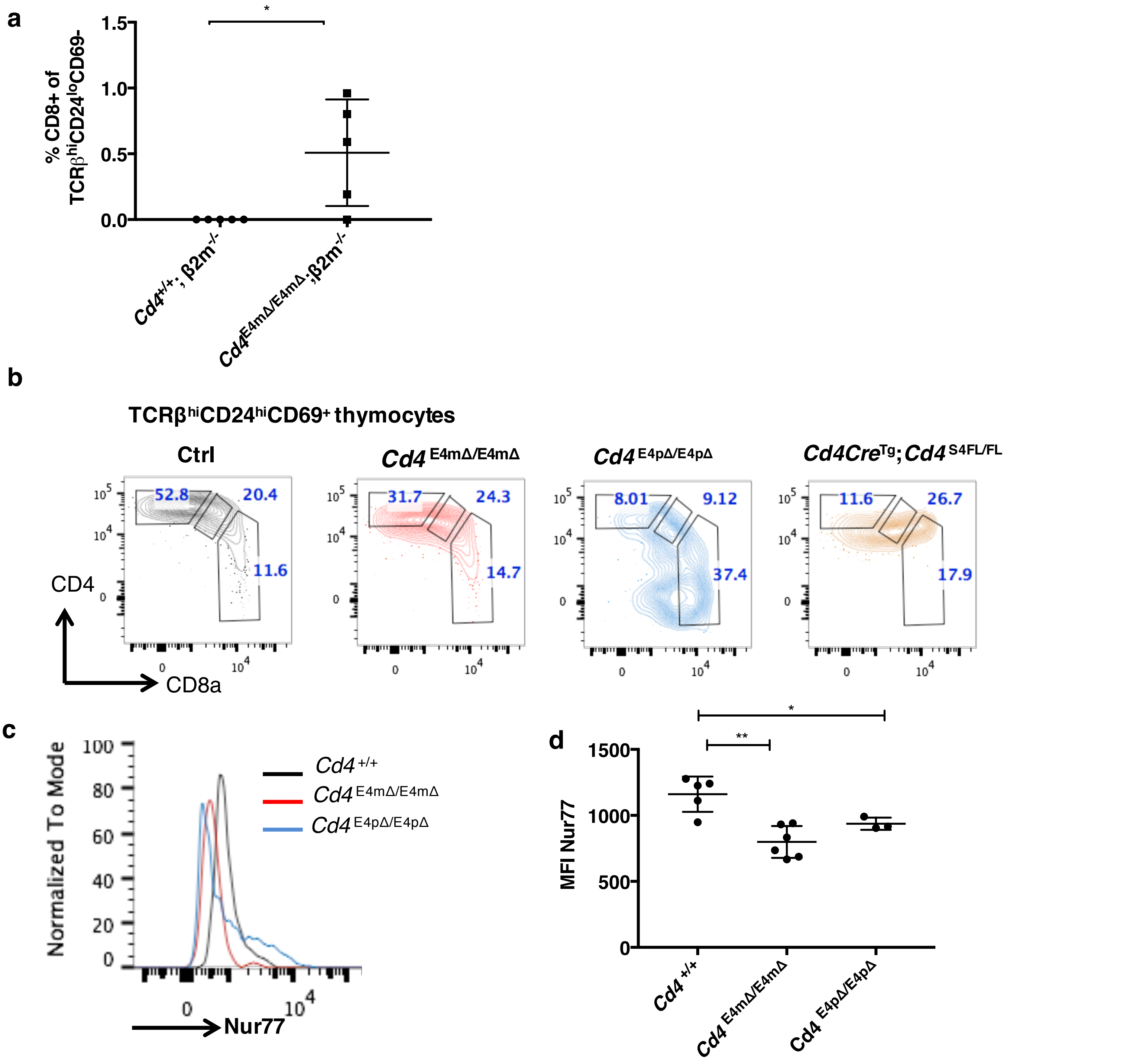

Supplementary Figure 4. ***Cd4*** **expression during positive selection is critical to prevent lineage re-direction. (a)** Proportions of CD8 SP thymocytes from *Cd4* $^{E4m\Delta/E4m\Delta}$; *β2m*$^{-/-}$ mice. Combined data with mice from 2 independent experiments. Mean ± SD. *p<0.05 (unpaired student *t* test). **(b)** CD4 and CD8 expression on recently selected thymocytes from mice with indicated genotypes. Numbers in the FACS plot quadrants indicate cell percentages. Representative results of 3 independent analyses with multiple mice. **(c)** Representative plots of intracellular Nur77 expression in recently selected CD4 SP TCR ^{hi}CD24^{hi}CD69^{+} thymocytes of mice with the indicated genotypes. Data are representative of 3 independent experiments with multiple mice. **(d)** Nur77 mean fluorescence intensity in thymocytes from mice with the indicated genotypes, as shown in representative data in (c). Combined mice from 2 independent experiments. Mean ± SD. *p<0.05, **p<0.005 (unpaired student *t* test).

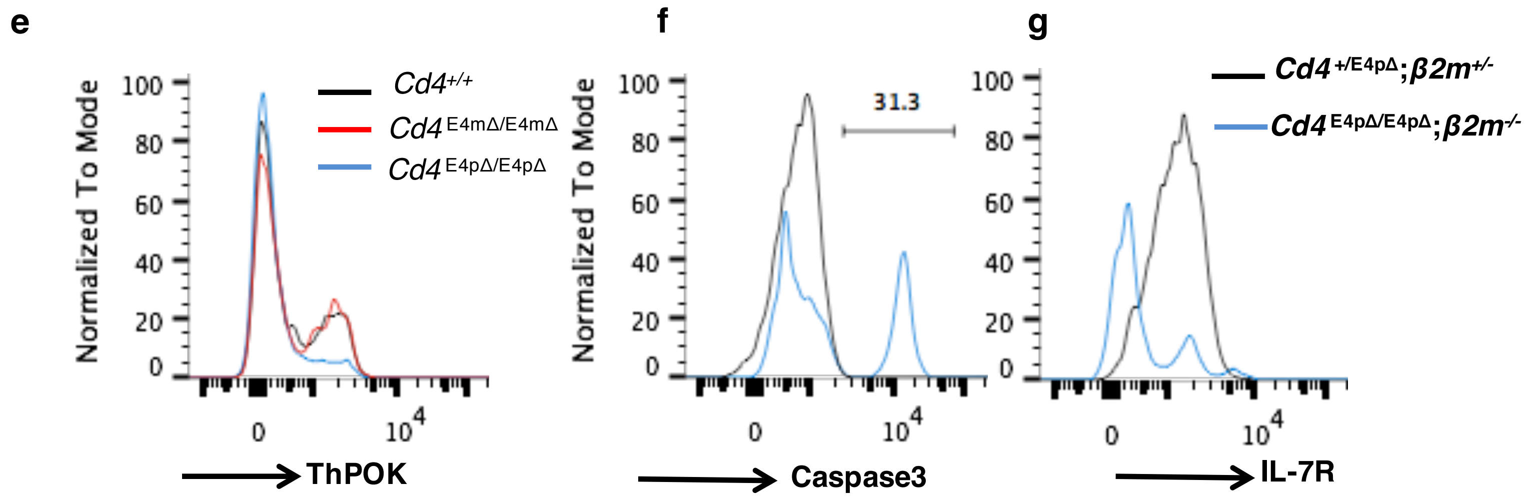

Supplementary Figure 4. *Cd4* expression during positive selection is critical to prevent lineage re-direction. (e) Intracellular ThPOK expression in recently selected TCR ^{hi}CD24^{hi}CD69^{+} thymocytes of mice with the indicated genotypes. Data are representative of 3 independent experiments with multiple mice. **(f)** Caspase-3 expression in TCR ^{hi}CD24^{lo} CD8 SP cells from *Cd4* $^{E4p\Delta/E4p\Delta}$; *β2m*$^{-/-}$ (blue) and control *Cd4* $^{E4p+/\Delta}$; *β2m*$^{+/-}$ mice (black). Experiment is representative of 2 mice. **(g)** IL-7R staining on TCR ^{hi}CD24^{lo} CD8 SP thymocytes from *Cd4* $^{E4p\Delta/E4p\Delta}$; *β2m*$^{-/-}$ mice (blue) and control *Cd4* $^{E4p+/\Delta}$; *β2m*$^{+/-}$ mice (black). Experiment is representative of 2 mice.

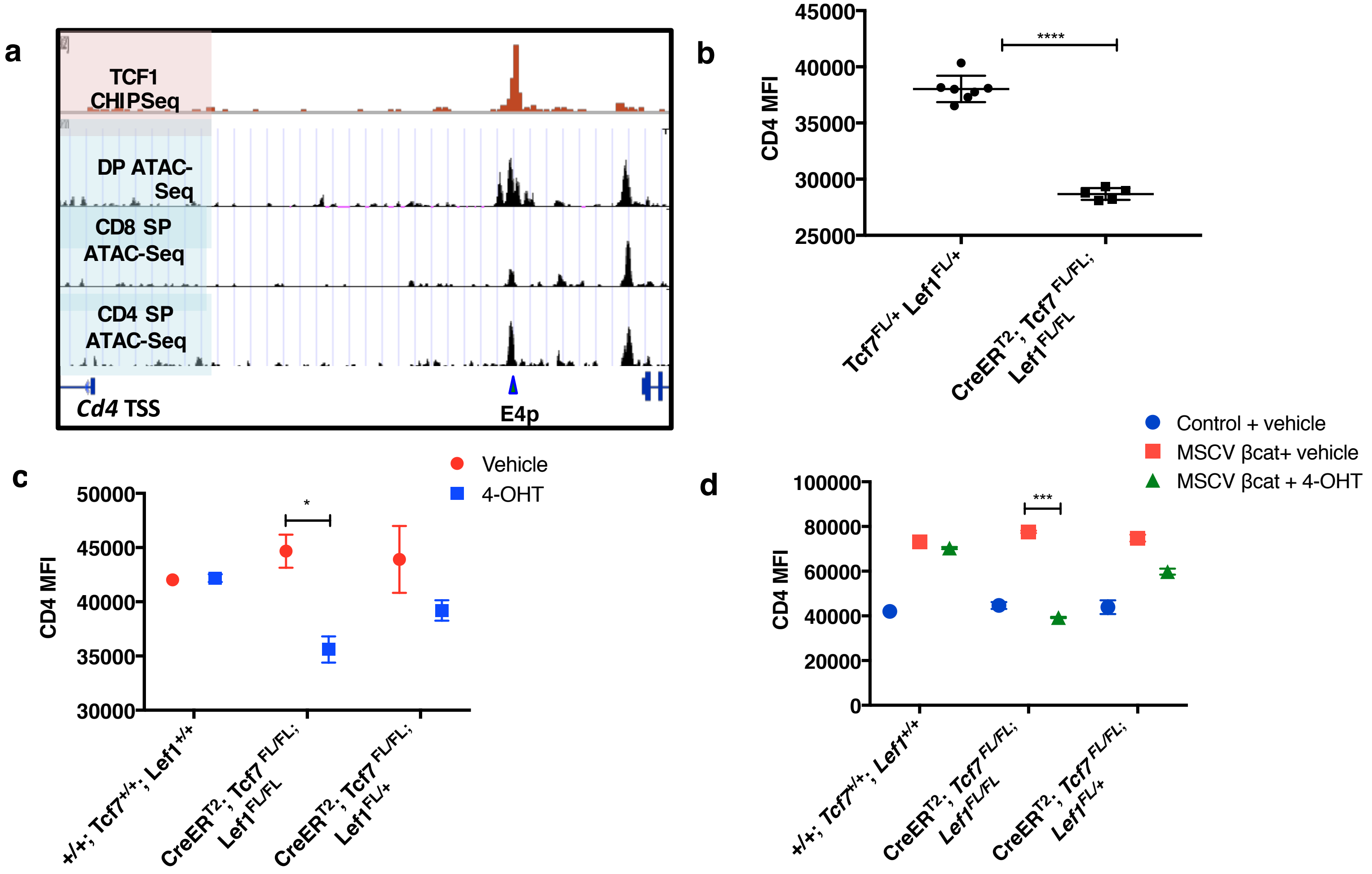

Supplementary Figure 5. ***Tcf7*** **and** ***Lef1*** **control** ***Cd4*** **expression in mature CD4$^+$ T cells in a -catenin-dependent manner**. **(a)** ChIP-seq dataset of TCF1 in thymocytes (Geo accession GSE46662) juxtaposed onto genome browser views of ImmGen ATAC-seq datasets from DP, CD4 SP and CD8 SP thymocytes. The location of E4p is indicated. **(b)** Graph showing the MFI quantification of CD4 on CD4$^+$ TCR $^+$ splenocytes of mice with the indicated genotypes treated with tamoxifen for 4 days, and isolated 4 days post last treatment. Data are from mice combined from 3 independent experiments. Mean $\pm$ SD. ****$p<0.0001$ (unpaired student *t* test). **(c)** Graph showing the quantification of CD4 MFI on activated T cells from the indicated genotypes. Naïve CD4$^+$ T cells from the respective mice were FACS sorted and activated in the presence of 4-hydroxytamoxifen or vehicle control for 24 h and analyzed 5 days post activation. Data are representative of 2 experiments done with technical replicates. Mean $\pm$ SD. *$p<0.05$ (unpaired student *t* test) **(d)** Graph showing the MFI quantification of CD4 on activated T cells from the indicated genotypes 5 days post transduction with MSCV IRES-GFP or MSCV beta-catenin-IRES-GFP vectors. Naïve CD4$^+$ T cells from the respective mice were FACS sorted and activated in the presence of 4-hydroxytamoxifen or vehicle control for 24 h prior to transduction. Data are representative of 2 experiments done with technical duplicates. Mean $\pm$ SD. ***$p<0.001$ (unpaired student *t* test)

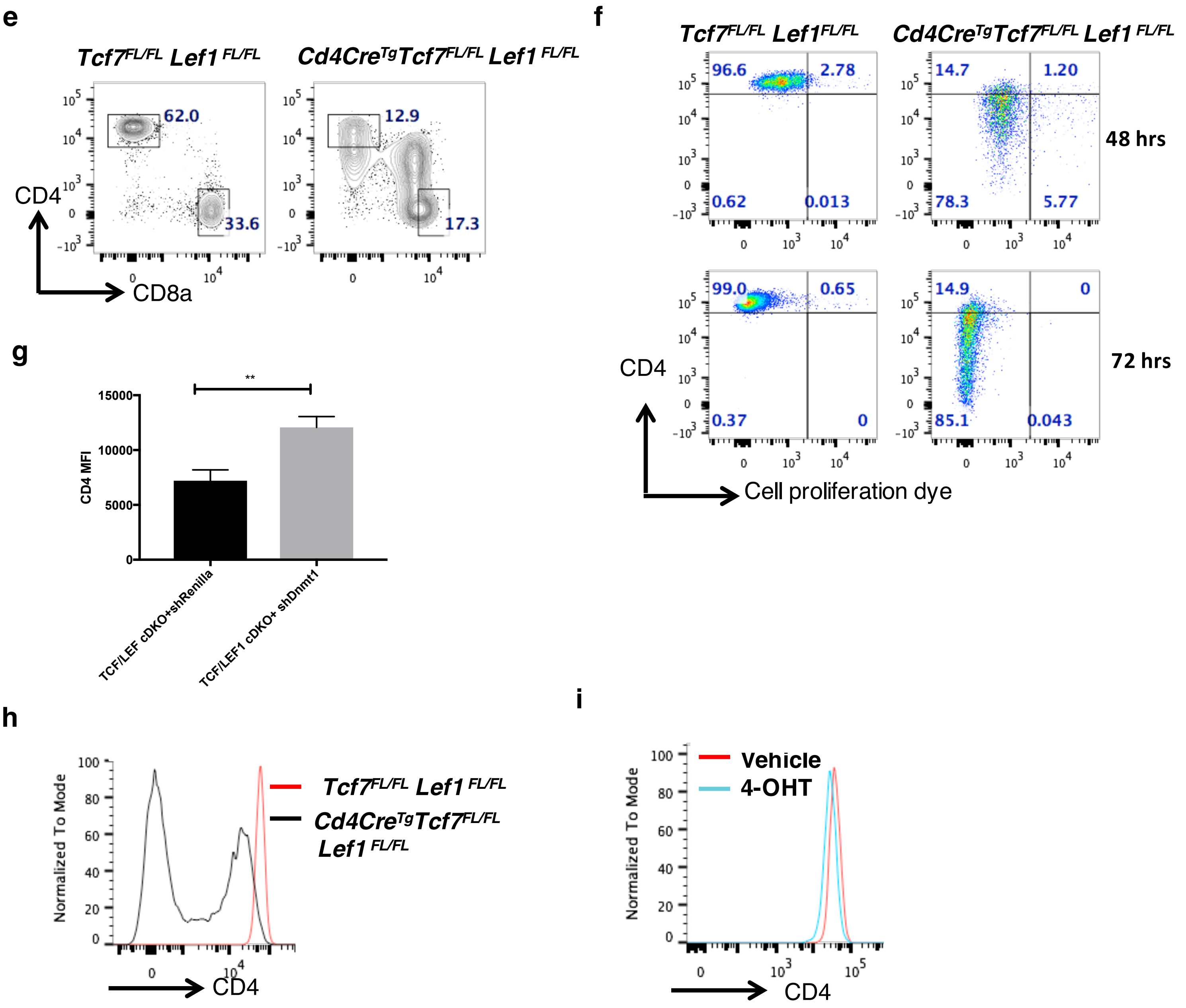

Supplementary Figure 5. ***Tcf7*** **and** ***Lef1*** **control** ***Cd4*** **expression in mature CD4$^+$ T cells in a -catenin-dependent manner**.**(e)** CD4 and CD8 expression on peripheral TCRβ$^+$CD19$^-$ cells from the spleen and lymph nodes of mice with the indicated genotypes. Data are representative of 2 mice. **(f)** CD4 expression following cell proliferation, monitored by cell proliferation dye-dilution at 48 and 72 h after *in vitro* activation with anti-CD3/anti-CD28. Sorted naive CD4$^+$ T cells from mice with indicated genotypes were labeled with cell proliferation dye before activation. Numbers in the indicated gate represent cell percentages. Data are representative of 2 mice. **(g)** CD4 MFI expression on activated CD4 T cells from *Cd4Cre*^{Tg}*Tcf7*$^{FL/FL}$ *Lef1*$^{FL/FL}$ mice transduced with shRNAs against *Renilla* (control) or *Dnmt1*. Data is from 2 mice with technical replicates. Mean $\pm$ SD. **p<0.01 (unpaired student *t* test). **(h)** Representative histogram overlay of CD4 expression on *in vitro* activated naïve CD4$^+$ T cells sorted from *Cd4Cre*^{Tg}*Tcf7*$^{FL/FL}$ *Lef1*$^{FL/FL}$ and *Tcf7*$^{FL/FL}$ *Lef1*$^{FL/FL}$ control mice. Naïve CD4 T cells were FACS sorted, activated with anti-CD3/anti-CD28 for 48 h, and analyzed 120 h post-activation. **(i)** Representative histogram overlay of CD4 expression on *in vitro* activated naïve CD4$^+$T cells from *CreERT*^{2} *Tcf7*$^{FL/FL}$ *Lef1*$^{FL/FL}$mice. Naïve CD4 T cells were FACS sorted and activated with anti-CD3/anti-CD28 for 48 h, in the presence of 4-hydroxytamoxifen or vehicle control for 24 h, and cells were analyzed 120 h post-activation.

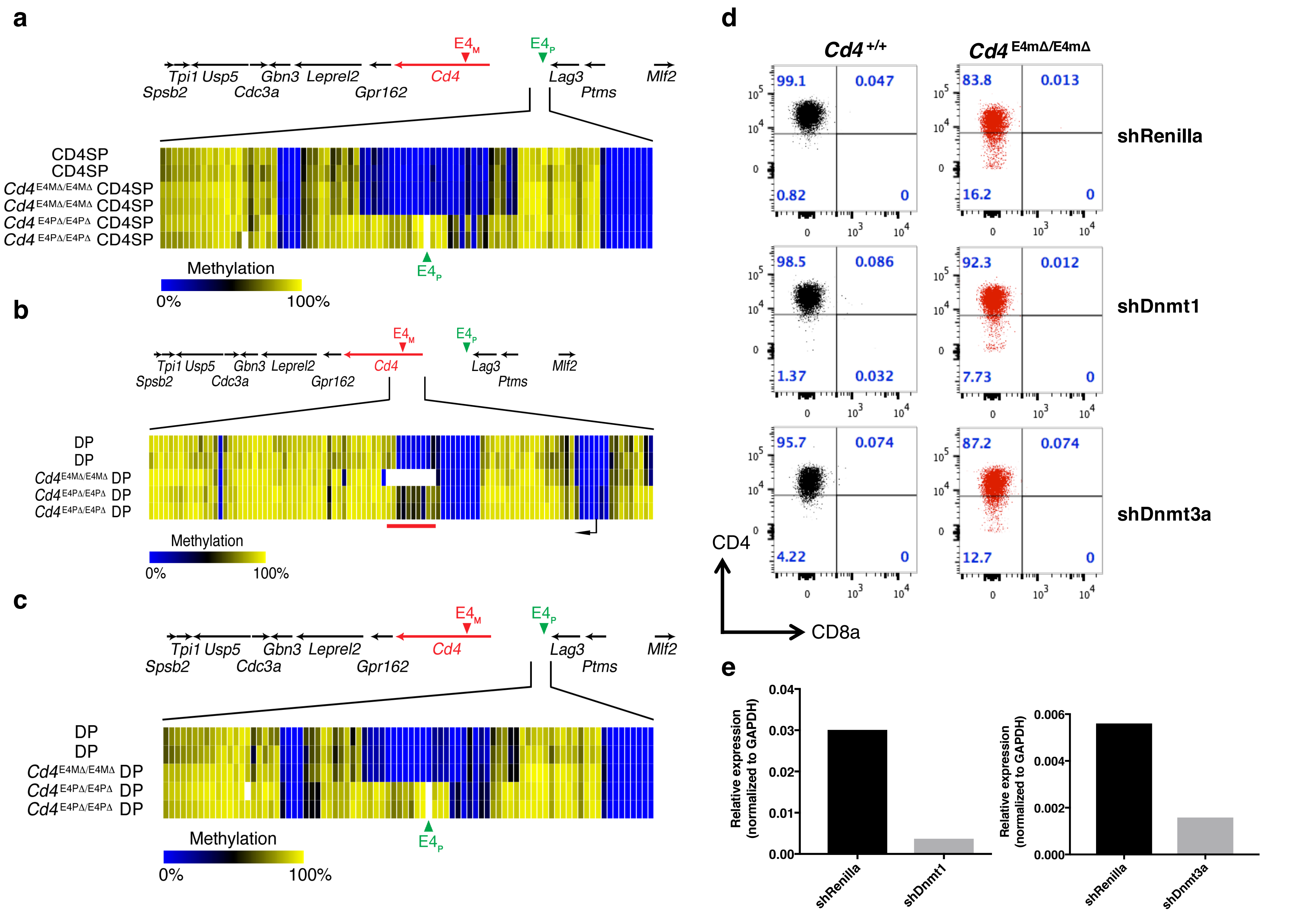

Supplementary Figure 6. E4m and E4p are both required for DNA demethylation necessary for stable *Cd4* expression. (a) Heatmaps depicting percentage CpG methylation from -9270bp to -15869bp relative to the *Cd4* TSS (Chr6:124847307-124853906; mm9). The approximate

location of the E4p region is indicated and the lone CpG within the proximal enhancer is indicated below the heat map (green arrow head). Replicates are from 2 independent mice. CATCH-seq was performed on sorted populations of TCRβ^{hi}CD24^{lo}CD69^{-}CD4^{+}CD8^{-} thymocytes. Data for *Cd4*$^{E4p\Delta/E4p\Delta}$ conditions were from previously published experiments with similar experimental conditions[9]. **(b,c)** Heatmaps depicting percent CpG methylation in WT DP, *Cd4*$^{E4m\Delta/E4m\Delta}$ DP and *Cd4*$^{E4p\Delta/E4p\Delta}$ DP thymocytes, In (b), CpGs were from +6200 to −669 relative to the *Cd4* TSS (Chr6:124832027-124838896; mm9). A red line underlines CpGs in E4m (indicated by the gap in E4m mutant mice), and a black arrow indicates the *Cd4* TSS. In (c), CpG methylation was monitored as in panel (a). Replicates are from 2 independent mice. CATCH-seq was performed on sorted populations of TCRβ^{lo}CD24^{hi}CD69^{-}CD4^{+}CD8^{+} thymocytes. Data for *Cd4*$^{E4p\Delta/E4p\Delta}$ conditions were from previously published experiments with similar experimental conditions[9]. **(d)** Effect of Dnmt knockdowns on CD4 expression on activated T cells from the indicated genotypes. *Cd4*$^{E4m\Delta/E4m\Delta}$ CD4^{+} T cells were stimulated *in vitro* for 24 h with anti-CD3 and anti-CD28 and transduced with retroviral vectors expressing either shRenilla, sh*Dnmt1* or sh*Dnmt3a*. GFP^{+} cells were analyzed 4 days post-transduction. Data are representative of 3 experiments performed with shDnmt1 and 2 experiments performed with shDnmt3a. **(e)** RT-qPCR analysis of *Dnmt1* (left) and *Dnmt3a* expression (right) after normalization to *Gapdh* expression in activated T cells from WT or *Cd4*$^{E4m\Delta/E4m\Delta}$ CD4^{+} T cells transduced with shRenilla, shDnmt1 or shDnmt3a. Transduced *Cd4*$^{E4m\Delta/E4m\Delta}$ GFP^{+} cells were sorted and analyzed at 4 days after transduction.

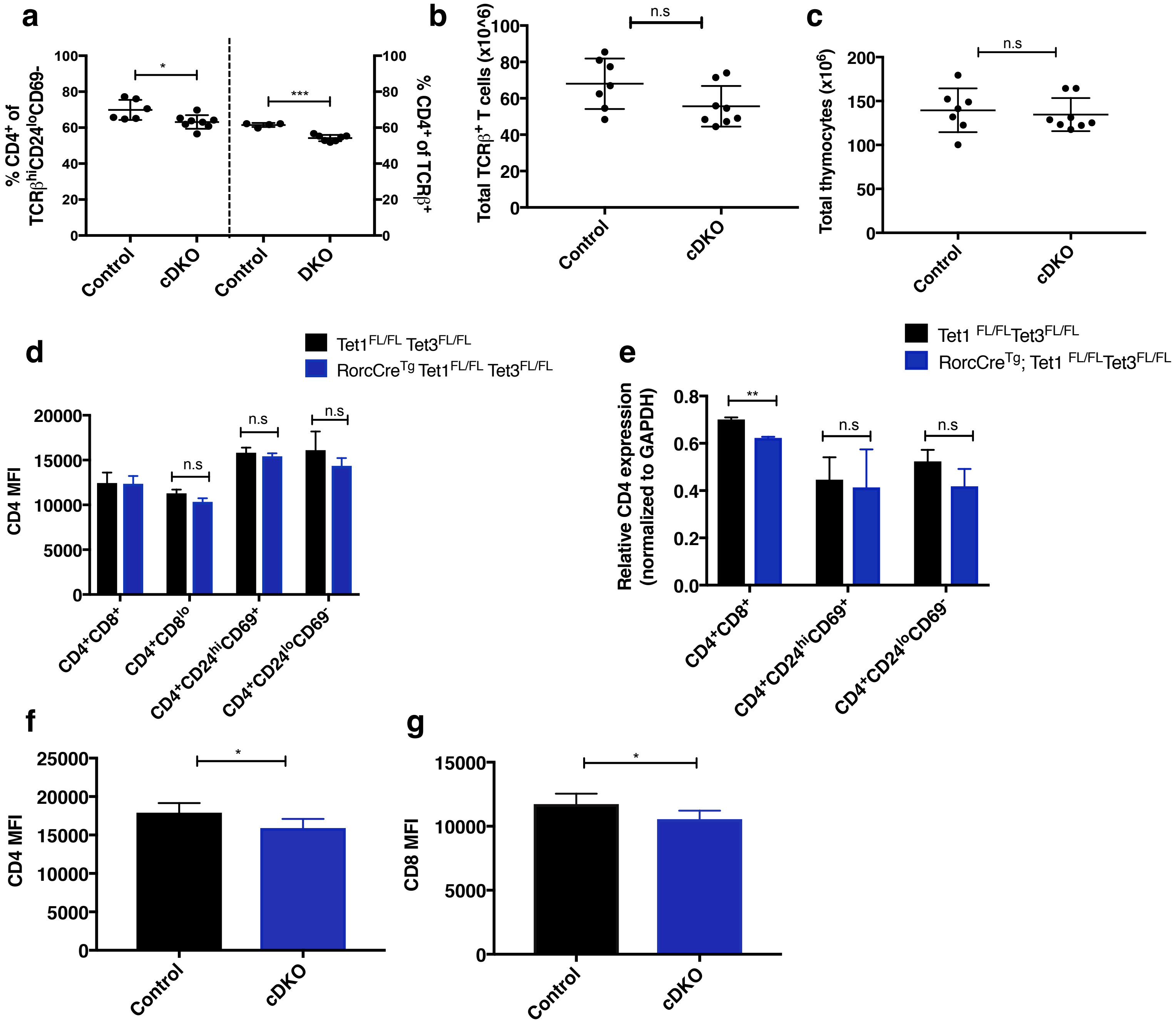

Supplementary Figure 7. TET1 and TET3 are required for E4m- and E4p-dependent demethylation of *Cd4*. (a) Proportions of CD4 SP T cells among thymic TCR ^{hi}CD24^{lo}CD69^{-} cells (left) and among TCR $^{+}$ cells from the lymph nodes and spleen (right) of control and *RorcCre*^{Tg}*Tet1*$^{FL/FL}$*Tet3*$^{FL/FL}$ mice. Mean ± SD. *p<0.05, ***p<0.001 (unpaired student *t* test). *RorcCre*^{Tg}*Tet1*$^{FL/+}$*Tet3*$^{FL/+}$ and *Tet1*$^{FL/FL}$ *Tet3*$^{FL/FL}$ mice were used as controls. **(b)** Total number of TCRβ^{+} T cells from the lymph nodes and spleen of control and *RorcCre*^{Tg}Tet1$^{FL/FL}$Tet3$^{FL/FL}$ mice. *RorcCre*^{Tg}*Tet1*$^{FL/+}$*; Tet3*$^{FL/+}$ and *Tet1*$^{FL/FL}$ *Tet3*$^{FL/FL}$ mice were used as controls. Mean ± SD. n.s p>0.05 (unpaired student *t* test) **(c)** Total number of thymocytes from control and *RorcCre*^{Tg}*Tet1*$^{FL/FL}$*Tet3*$^{FL/FL}$ mice. *RorcCre*^{Tg}*Tet1*$^{FL/+}$*; Tet3*$^{FL/+}$ and *Tet1*$^{FL/FL}$ *Tet3*$^{FL/FL}$ mice were used as controls. Mean ± SD. n.s p>0.05 (unpaired student *t* test). **(d)** Quantification of CD4 MFI on indicated subsets of cells from *RorcCre*^{Tg} *Tet1*$^{FL/FL}$ *Tet3*$^{FL/FL}$ mice and littermate controls. Mean ± SD. n.s p>0.05, (unpaired student *t* test); data are representative of 2 experiments, with 3 mice in each group per experiment. **(e)** RT-qPCR analysis of *Cd4* mRNA levels in the indicated subsets of cells from *RorcCre*^{Tg} *Tet1*$^{FL/FL}$ *Tet3*$^{FL/FL}$ mice and littermate controls. Mean ± SD n.s p>0.05, (unpaired student *t* test); data are for 3 mice in each group. **(f-g)** Quantification of CD4 **(f)** or CD8 MFI **(g)** on naïve TCRβ^{+}CD62L^{hi} CD44^{Lo}CD4^{+} T cells from *RorcCre*^{Tg} *Tet1*$^{FL/FL}$ *Tet3*$^{FL/FL}$ mice and littermate controls. Mean ± SD. * p<0.05, (unpaired student *t* test) and data is representative of 2 experiments, with 3 mice in each group per experiment.

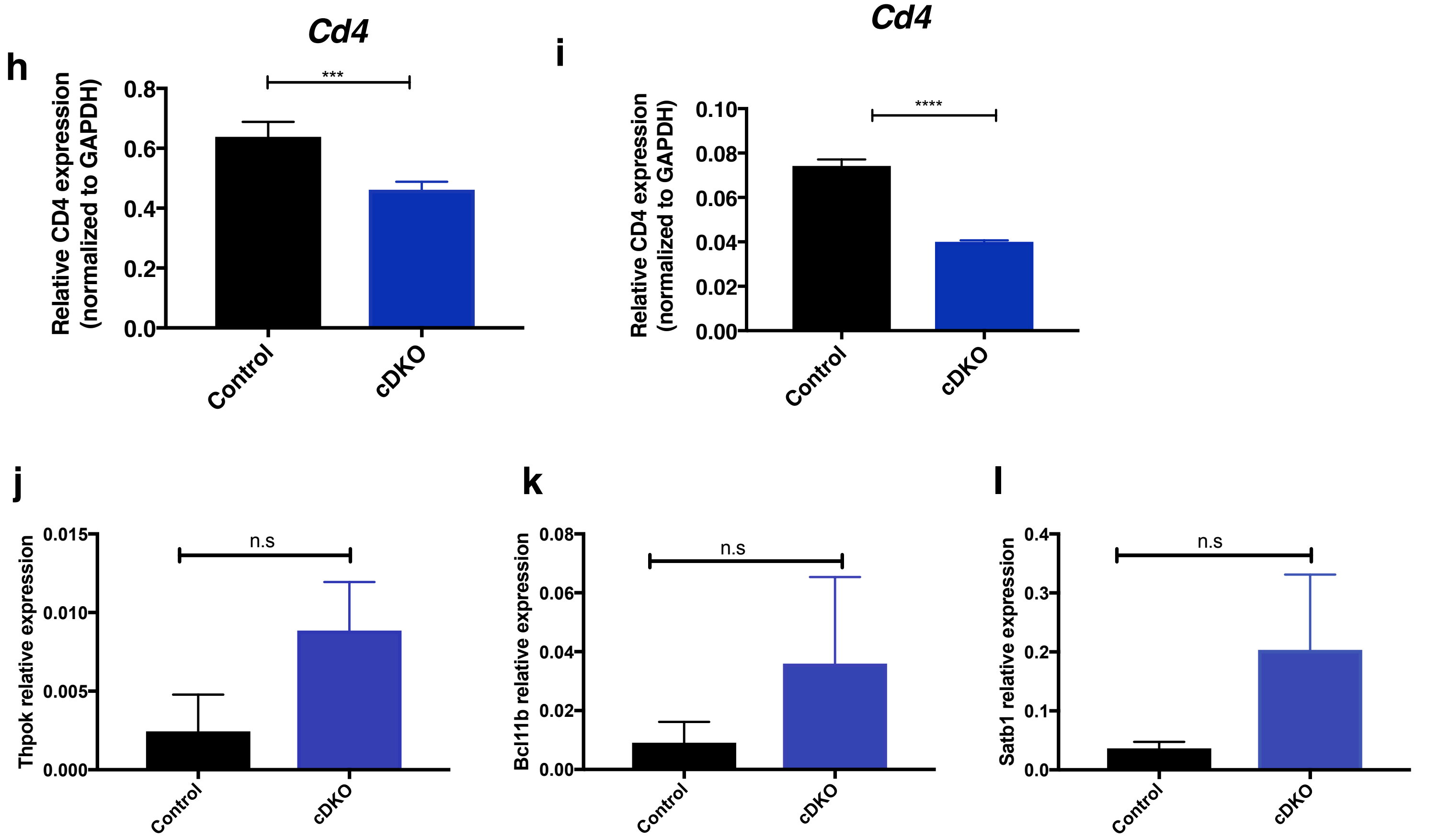

Supplementary Figure 7. TET1 and TET3 are required for E4m- and E4p-dependent demethylation of *Cd4*. (h) RT-qPCR analysis of *Cd4* mRNA levels in naïve TCRβ^{+}CD62L^{hi} CD44^{Lo}CD4^{+} T cells from *RorcCre^{Tg} Tet1$^{FL/FL}$ Tet3$^{FL/FL}$* mice and littermate controls. Mean $\pm$ SD *p<0.05, (unpaired student *t* test) and data are shown for 3 mice in each group. **(i-l)** RT-qPCR analysis of *Cd4, Thpok, Bcl11b and Satb1* mRNA levels in *in vitro* activated CD4+ T cells from *RorcCre^{Tg} Tet1$^{FL/FL}$ Tet3$^{FL/FL}$* mice and littermate controls. Mean $\pm$ SD *p<0.05, (unpaired student *t* test) and data are shown for 3 mice in each group. Cells were sorted 5 days post activated and qPCR analysis were done on CD4^{lo} sorted fractions from *RorcCre^{Tg} Tet1$^{FL/FL}$ Tet3$^{FL/FL}$* cells and CD4^{hi} fractions from control cells.

SUPPLEMENTARY TABLE 1

**qPCR Primers**	**Sequence**
CD4 Fwd	TGTCACTCAAGGGAAGACGC
CD4 Rev	CGAAGGCGAACCTCCTCTAA
Thpok Fwd	CCCTGCTCGAGTTTGCTTAC
Thpok Rev	CTCGCTCACAGTCATCCTCA
Tet3 Fwd	AACGGCTGCAAATATGCTCG
Tet3 Rev	TCCTCCTCCTTCGGATTGTCT
Tet1 Fwd	CCTAGTCTCCATGAGCTCCC
Tet1 Rev	AACAAAGCCCCTGTGCTATTTG
Tet2 Fwd	AACCTGGCTACTGTCATTGCTCCA
Tet2 Rev	ATGTTCTGCTGGTCTCTGTGGGAA
Cd4^{E4p} Fwd	AGCCCCACCCTAAGATGAAG
Cd4^{E4p} Rev	CCTTTGGAGAAACTGCCAG
Thpok Fwd	CCCTGCTCGAGTTTGCTTAC
Thpok Rev	CTCGCTCACAGTCATCCTCA
Bcl11b Fwd	ATCCGTGAAGCCCAGGAATG
Bcl11b Rev	GGTGGAGAAAGACCTGGAGC
Satb1 Fwd	TCGTTTCAGTTCATCCCGCA
Satb1 Rev	AGCCCAGCAGTCCTTAAACC

**SgRNA**	**Sequence (minus PAM site)**
Cd4$^{E4m\Delta/E4m\Delta}$ 5'	AAGCCAGGCTACTTGTTTAC
Cd4$^{E4m\Delta/E4m\Delta}$ 3'	ACTGACACACCCGCTCATCA

**Genotyping primers**	**Sequence**
Cd4$^{E4m\Delta/E4m\Delta}$ Fwd	CTGTGAAGGGTGGTTGTTGC
Cd4$^{E4m\Delta/E4m\Delta}$ Rev	GTTTCCTGCTGTCCCTACCC

**Capture C Biotinylated probes**	**Sequence**
E4m 5'end	Gatctaaattcacgtagctctgataaagatgctggaggaacgagggcattcctggctcttccccagagggtaggcactttctctccctcactgtacaaccttggagtctgtctccagctc
E4m 3'end	tttggccttcagctccttggatgtcttcctctgagctgcagcctttagtgttctcttccccttggagtgccccctatctcctctttctcccctttcctctcctcagatgccaccaagatc
S4 5'end	Gatccatcaacacatgacaagaacatgtcagaaggagaggcataggaatgaagagactctagaaggcaagatacatgcagagaactcaacaccaaaagaacaaagagagggaccacacaa
S4 3'end	gcctcttcctagaactggttgaccgtggggaagactctgtacatagaggcaggcctcttctgtgagggcctcggattgaaagggaaaactggttctaggtatctggctcctcggaggatc
